# The Markyt visualisation, prediction and benchmark platform for chemical and gene entity recognition at BioCreative/CHEMDNER challenge

**DOI:** 10.1093/database/baw120

**Published:** 2016-08-19

**Authors:** Martin Pérez-Pérez, Gael Pérez-Rodríguez, Obdulia Rabal, Miguel Vazquez, Julen Oyarzabal, Florentino Fdez-Riverola, Alfonso Valencia, Martin Krallinger, Anália Lourenço

**Affiliations:** 1 ESEI - Department of Computer Science, University of Vigo, Ourense, Spain; 2 Small Molecule Discovery Platform, Molecular Therapeutics Program, Center for Applied Medical Research (CIMA), University of Navarra, Pamplona, Spain; 3 Structural Computational Biology Group, Structural Biology and BioComputing Programme, Spanish National Cancer Research Centre, Madrid, Spain; 4 CEB - Centre of Biological Engineering, University of Minho, Braga, Portugal

## Abstract

Biomedical text mining methods and technologies have improved significantly in the last decade. Considerable efforts have been invested in understanding the main challenges of biomedical literature retrieval and extraction and proposing solutions to problems of practical interest. Most notably, community-oriented initiatives such as the BioCreative challenge have enabled controlled environments for the comparison of automatic systems while pursuing practical biomedical tasks. Under this scenario, the present work describes the Markyt Web-based document curation platform, which has been implemented to support the visualisation, prediction and benchmark of chemical and gene mention annotations at BioCreative/CHEMDNER challenge. Creating this platform is an important step for the systematic and public evaluation of automatic prediction systems and the reusability of the knowledge compiled for the challenge. Markyt was not only critical to support the manual annotation and annotation revision process but also facilitated the comparative visualisation of automated results against the manually generated Gold Standard annotations and comparative assessment of generated results. We expect that future biomedical text mining challenges and the text mining community may benefit from the Markyt platform to better explore and interpret annotations and improve automatic system predictions.

**Database URL**: http://www.markyt.org, https://github.com/sing-group/Markyt

## Introduction

A contemporary, well-recognized challenge of Bioinformatics is to develop specialized methods and tools that enable the systematic and large-scale integration of scientific literature, biological databases and experimental data (1–3). These tools have the potential of considerably reducing the time of database curation and enabling on demand and highly specialized access to literature and database contents (4–6).

Initiatives such as BioCreative have been brewing the development of such tools, by providing annotated literature corpora (7, 8) and enabling the controlled comparison of systems performing the automated recognition of biomedical entities of practical interest (9, 10).

**Figure 1.**
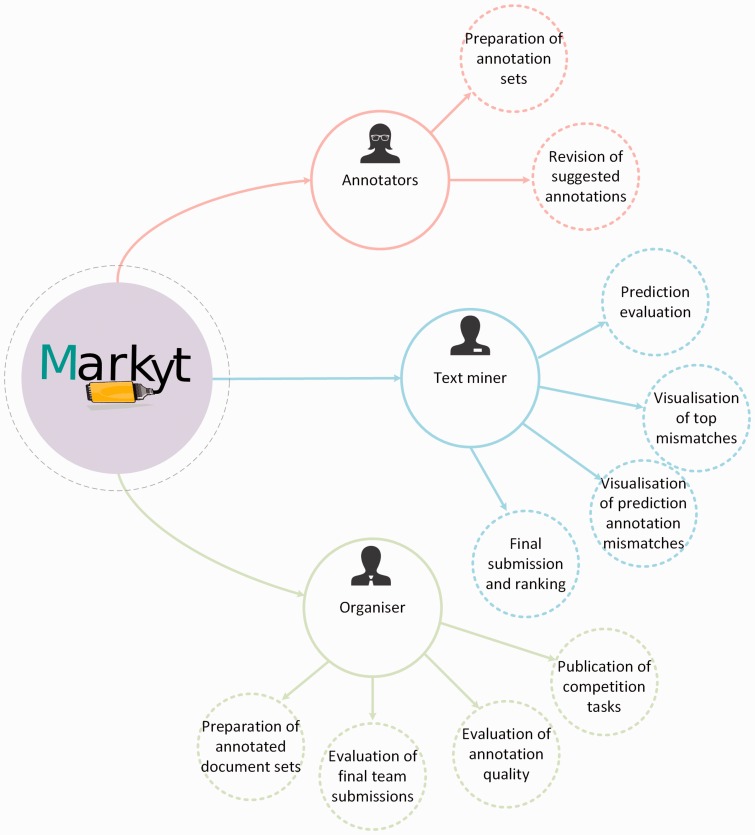
Main use cases of Markyt at BioCreative V CHEMDNER patents challenge. The system helped the organizers and annotators to prepare the annotated document sets, supported the work of text miners while tuning up their systems, and enabled the evaluation and ranking of final predictions.

Under this scenario, the latest BioCreative V CHEMDNER patents challenge (11, 12), which addressed the automatic extraction of chemical and biological data from medicinal chemistry patents, aimed to go a step forward and integrated new computational means to optimize the efforts of both annotators and participants. Specifically, a new Web-based visualisation, prediction and benchmark platform was devised in support of the chemical and gene entity recognition tasks (Figure 1). The CHEMDNER challenge organizers used this platform, named Markyt, to prepare the annotated document sets and to evaluate the predictions of the participating systems. The platform provided a *user-friendly document visualisation* environment, where human annotators could manage annotation sets and project administrators could evaluate the quality of the annotations throughout the annotation process. On the other hand, Markyt offered participants the possibility of *evaluating their predictions* on different annotated document sets, so that they could explore prediction–annotation mismatches and acquire insights on possible system improvements. Also, it was used for the final submission of task predictions and the automated scoring of the teams. Currently, the platform is *supporting post-workshop prediction evaluation*, i.e. any developer can now test their software against CHEMDNER corpora and compare their results with those obtained in the competition.

The aim of this paper is to describe the operation of Markyt platform for chemical and gene entity recognition at BioCreative/CHEMDNER challenge and its support to the broader use of the challenge’s resources by text mining developers. The next sections present the architectural design of the platform and show how the platform was utilized by the different users throughout the challenge.

## Materials and Methods

This section describes the main features of the Markyt platform for the visualisation, prediction and benchmark of chemical and gene entity recognition in medicinal chemistry patents under the scope of the BioCreative V CHEMDNER patents challenge (11, 12).

**Figure 2.**
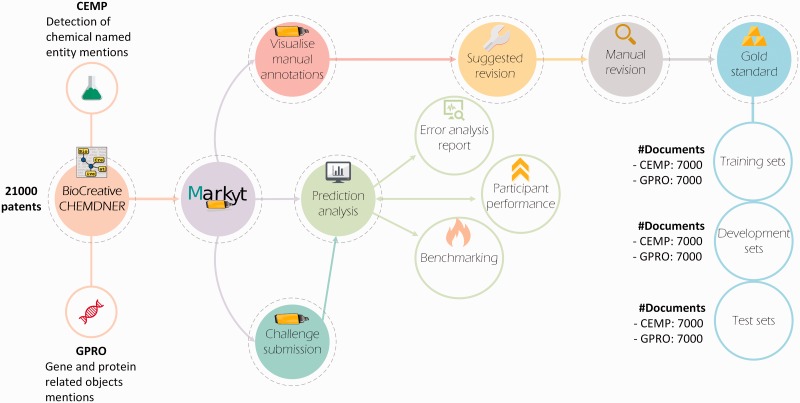
The Markyt platform for chemical and gene entity recognition at BioCreative/CHEMDNER challenge. Markyt was used in CEMP and GPRO tasks, supporting the preparation of training, development and test sets and enabling controlled prediction evaluation and benchmarking.

As illustrated in Figure 2, Markyt platform was used to revise the manual labelling of the datasets for the chemical entity mention in patents (CEMP) and gene and protein related object (GPRO) tasks, which entailed the detection of chemical named entity mentions and mentions of gene and protein related objects in patent titles and abstracts, respectively. The annotated document sets used for training and development were produced with the intent of supporting the improvement of the automatic prediction tools enrolled in the challenge. Conversely, the test sets were used in the controlled comparison of the performance of the participating systems. Markyt enabled both the analysis of automatic predictions by participants and the controlled comparison of the performance of the various systems.

Next, we detail the main aspects of the platform, in terms of software architecture, challenge requirements and user–system interaction.

### General architecture

Markyt is a Web-based platform that was initially devised for the management of multi-user iterative annotation projects (13). Supported by open-source consolidated technologies and presents a modular design, which enabled the development of new modules in response to CHEMDNER’s requirements.

The platform follows the Model-View-Controller architectural pattern and was developed using the open source CakePHP Web framework (14). At the core of its architecture are consolidated Web technologies. PHP programming language (version 5.5) and the MySQL database engine (version 5.1.73) support the server side operations. HTML5 (http://www.w3.org/TR/html5/) and CSS3 technologies (http://www.css3.info/) provide for common interface features. Browser-independent implementation of common DOM range and selection tasks is achieved using the Rangy library (http://code.google.com/p/rangy/). Finally, Ajax and JQuery (http://jquery.com/) technologies help in user–system interaction, such as document manipulation, event handling, animation and efficient use of the network layer.

### Annotation environment

The annotation environment of Markyt supports the customized deployment of multi-user and multi-round annotation projects. It provides an intuitive interface to visualize and edit annotated document sets, keeps track of multiple rounds of annotation and allows the comparison of annotation quality across rounds and among annotators. At the technical level, this tool manipulates documents in HTML format and encoded using UTF-8. Annotation classes are represented by HTML class labels and customized to meet the specifications of the project. Moreover, Markyt allows three main types of annotations: manual, i.e. performed by a human annotator; automatic, i.e. originating from an automatic recognition system; and semi-automatic, i.e. automatic annotation of text fragments that are similar to a manual annotation.

Two annotation projects were created for the CHEMDNER challenge, one for the CEMP task and another one for the GPRO task, so that annotators could manage each task independently and adequately. Furthermore, the CEMP and GPRO annotation projects were configured according to the following annotation guidelines:

- Annotations could include single or multiple words, or even partial fragments of a word.
- Annotation class is unique and exclusive so that the annotations can only belong to one annotation class.
- Nested annotations are not allowed, i.e. the offsets of one annotation cannot be between the offsets of another annotation in the same document.
- CEMP entity mentions were divided into eight classes: SYSTEMATIC, TRIVIAL, FAMILY, FORMULA, ABBREVIATIONS, IDENTIFIERS, MULTIPLE and NO CLASS (details can be found at http://www.biocreative.org/media/store/files/2015/cemp_patent_guidelines_v1.pdf).
- GPRO entity mentions were classified into four classes: NESTED MENTIONS, IDENTIFIER, FULL NAME and ABBREVIATION (details can be found at http://www.biocreative.org/media/store/files/2015/gpro_patent_guidelines_v1.pdf).

Project administrators and human annotators were instructed on how to operate Markyt annotation environment and were assisted throughout the annotation process. Markyt was used primarily as a visualisation and editing tool, which helped project administrators and annotators to discuss the quality of the annotations in the different document sets.

Under this scenario, the automatic annotation recommendation module of Markyt played an important part in helping reduce the number of false negative mentions. More specifically, this annotation module was utilized to produce annotation recommendations based on the annotation history of the project. That is, the tool detected unlabelled text mentions that are exact, case insensitive matches of manual expert annotations and prompted recommendations to be manually revised. Manual annotations and automatic annotations were made visually distinguishable to make human inspection easier. Likewise, the human curator was only asked to remove the automatic recommendations that were incorrect. At the end of the manual revision, all remaining automatic annotations were accepted.

### Prediction analysis

CHEMDNER released training and development annotated document sets that participants could use to improve the performance of their automatic prediction tools and a blinded test set for which participants had to submit predictions to be evaluated against manual annotations. Markyt platform provided participants with an analytical environment for evaluating their predictions for the different gold standards.

In previous editions of the challenge, the organizers made available an evaluation script to score the predictions (http://www.biocreative.org/resources/biocreative-ii5/evaluation-library/). However, it was not straightforward to identify which terms were often missed or particular scenarios where the algorithm would output false positive predictions. Such exploration was either conducted in a manual way, which was time consuming, or supported by in-house software, which implied additional programming effort for most participants.

Markyt analytical environment aimed to bridge this gap and equip the teams with means to calculate prediction scores and explore the most important prediction–annotation mismatches without any additional programming costs. Therefore, it provides the calculation of micro- and macro-average standard performance statistics, such as precision, recall and F-score (15, 16). Furthermore, it enables the examination of annotation mismatches, i.e. false positive (FP) and false negative (FN) annotations. Three main statistics are examined: *false negative* (FN) results corresponding to incorrect negative predictions (i.e. cases that were part of the gold standard, but missed by the automatic system), *false positive* (FP) results being cases of incorrect positive predictions (i.e. wrong results predicted by the automatic system that had no corresponding annotation in the gold standard) and *true positive* (TP) results consisting of correct positive predictions (i.e. correct predictions matching exactly with the gold standard annotations).

Correspondingly, recall is the percentage of correctly labelled positive results over all positive cases, i.e. it is a measure of the ability of a system to identify positive cases.

(1) $$recall=\;\frac{TP}{TP+FN}$$

Precision is the percentage of correctly labelled positive results over all positive labelled results, i.e. it is a measure of the reproducibility of a classifier of the positive results.

(2) $$precision=\;\frac{TP}{TP+FP}$$

And, the F-score is the harmonic mean between precision and recall.

(3) $$F-score=2*\frac{precision\times recall}{precision+recall}$$

Partial hits, i.e. predictions that only in part overlapped with the manually defined gold standard annotations, were not taken into account in the analyses. Micro-average statistics were calculated globally by counting the total true positives, false negatives and false positives. Conversely, macro-average statistics were calculated by counting the true positives, false negatives and false positives on a per-document basis and then averaged across documents.

### Benchmarking

CHEMDNER participants could submit a total of five runs per task for final evaluation. The micro-averaged recall, precision and F-score statistics were used for final prediction scoring, and F-score was used as main evaluation metric.

Furthermore, Markyt analytical environment supported the examination of the statistical significance of each prediction with respect to the other final submissions by means of bootstrap resampling simulation, in a similar way to what was done in the previous CHEMDNER challenge (9, 17). This statistical analysis was done for both the CEMP and GPRO tasks by taking 2500 bootstrapped samples from all the documents in the test sets (a total of 7000 documents in each set) that had annotations. The micro-average F-scores for each team on each sample were calculated and these 2500 resampled results were then used to calculate the standard deviation of the F-score of each team (SDs). Teams were grouped based on statistically significant difference (at two SD) between results.

## Results

### Management of annotation projects and gold standard preparation

The CHEMDNER challenge involved the annotation of a total of 21 000 medicinal chemistry patents (11, 12). The patents came from the following agencies: the World Intellectual Property Organization (WIPO), the European Patent Office (EPO), the United States Patent and Trademark Office (USPTO), Canadian Intellectual Property Office (CIPO), the German Patent and Trade Mark Office (DPMA) and the State Intellectual Property Office of the People's Republic of China (SIPO).

CEMP and GPRO tasks were supported by the same document sets, but annotation was independent and complied with the guidelines established by the organizers for each task. Details on task guidelines can be found at http://www.biocreative.org/media/store/files/2015/cemp_patent_guidelines_v1.pdf.

**Figure 3.**
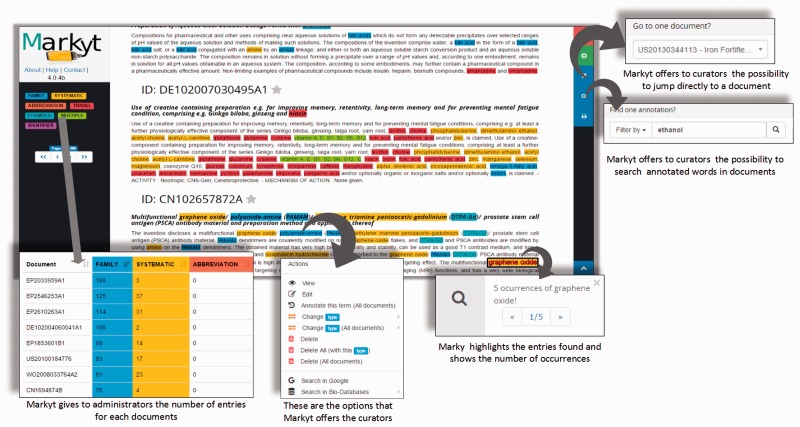
Some features of the Markyt document manual annotation environment. Examples were taken from one of the CEMP document sets and illustrate the visualisation of document contents and existing annotations, distribution of annotation per class, editing features, document search and annotation search.

As Figure 3 illustrates, Markyt annotation environment was used to manage the manual annotations in the document sets. Annotators were able to create, edit or delete annotations, navigate to specific documents or search for matches of a particular annotation. The task-dependent annotation types were defined at project configuration and colour tagged for immediate visual perception. For example, annotations of systematic chemical names in CEMP sets were tagged in yellow.

Besides providing basic means of text annotation, Markyt helped in minimising typical errors in repetitive tasks. For example, the annotator could apply the same operation to multiple inter-document occurrences of the marked text fragment. Markyt also allowed the search of documents containing a given annotation, and navigation to specific documents, which are operations suggested by the annotators as means to expedite annotation revision.

**Figure 4.**
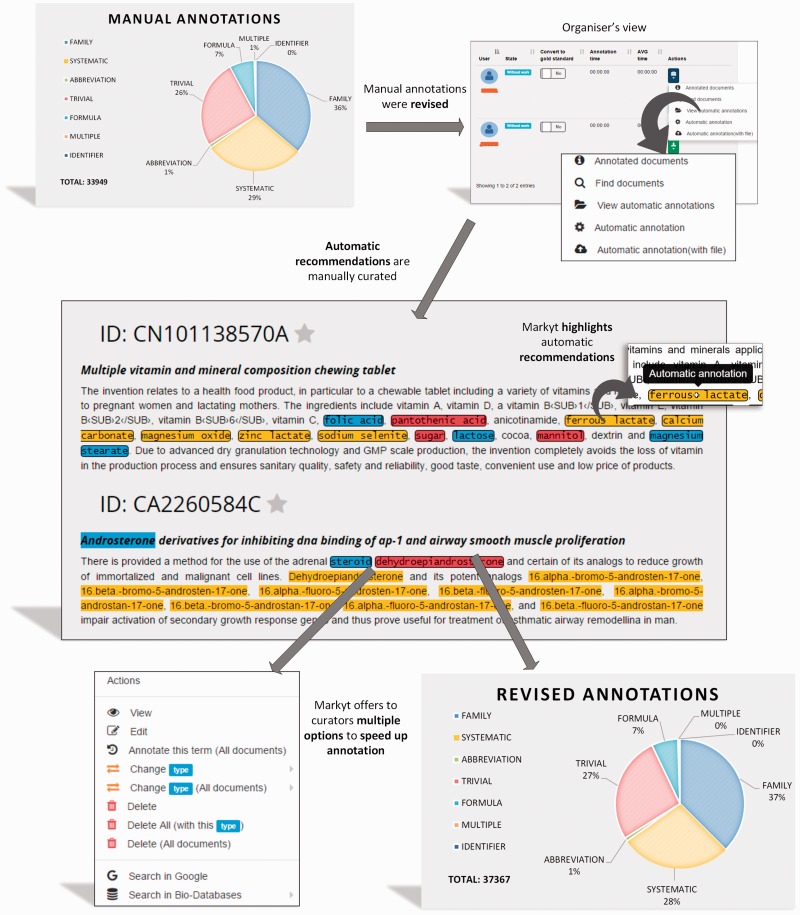
Semi-automated revision workflow of the CHEMDNER test set. The manually annotated document set is enriched with automatic annotation recommendations to be revised by the experts. Recommendations are based on unlabelled text mentions that match manual annotations. Annotators were required only to edit or eliminate non-qualifying recommendations.

Later, CEMP and GPRO test sets were submitted to an additional semi-automatic process of annotation (Figure 4). The manual annotations were used as ground truth by the *automatic recommendation module* of Markyt. That is, any text fragment matching one of these annotations and without an annotation was treated as a potential miss.

The annotation environment presented an integrated view of these recommended annotations together with the manual annotations to simplify manual expert revision. Automatic annotations were visually differentiated by the use of bordered marks. Annotators took advantage of the search and navigation features to inspect recommendations. Specially, annotators were instructed to edit or eliminate recommendations as considered appropriate. No manual action was required for accepting annotation recommendations, as this operation was performed automatically at the end of the revision round.

### Prediction analysis

**Figure 5.**
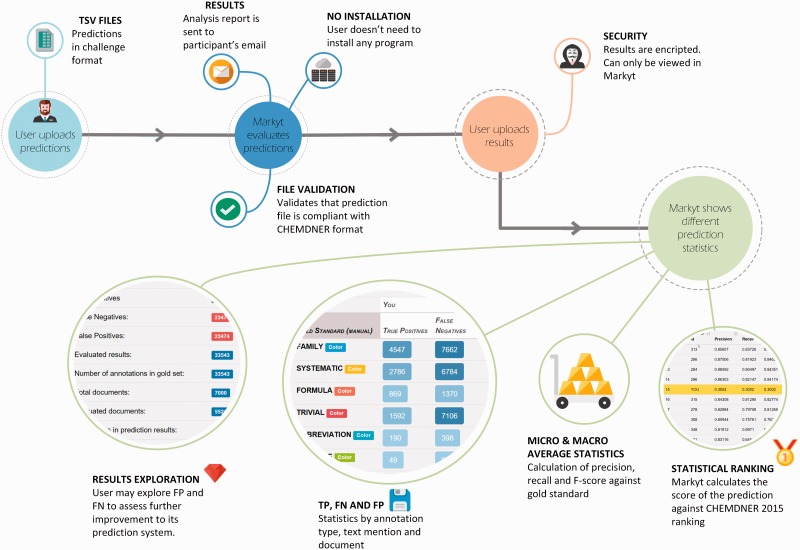
Markyt environment for prediction analysis. Teams could upload an unlimited number of predictions against the competition data sets. Markyt provided common performance metrics as well as details on false positives and false negatives. Final submissions were ranked based on micro averaged F-score.

Markyt measured the performance of the competing systems by comparing their predictions for gold standards (Figure 5). During the challenge, analysis was performed on demand by the participants. At the end of the challenge, this analysis was executed by the organizers as part of the automatic comparison and scoring of all competing teams.

Access to challenge participants was made simple. Using challenge credentials, the participant could submit prediction files (compliant with the CHEMDNER predictions file format, which is exemplified in the text data set available at http://www.biocreative.org/media/store/files/2015/CHEMDNER_TEST_TEXT.tar.gz). Markyt would perform the analysis and send the results encrypted via email (to avoid unnecessary wait when processing a large number of predictions). Then, the participant could access the system and visualize the prediction results privately.

**Figure 6.**
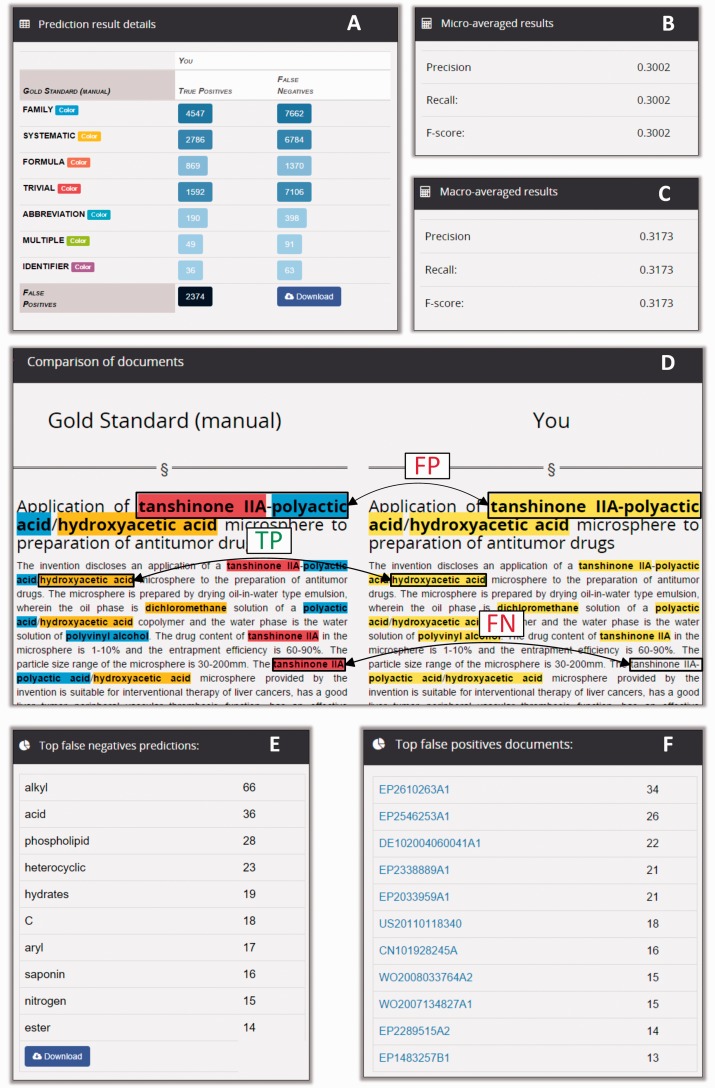
Snippets of the information displayed in a CEMP prediction analysis report. The report shows the distribution of true positives and false negatives per annotation type (A), and performance is described in terms of macro- and micro-averaged precision, recall and F-score statistics (B and C). The tool enables the exploration of prediction matches and mismatches in individual documents (D). Also, it enables the inspection of the most frequent misclassifications (E) and the documents with more mismatches (F).

The analysis report consisted of precision, recall and F-score statistics, a table with the distribution of true positive and false negative results per annotation class, and tables listing the top false positive and top false negative predictions (Figure 6). The micro-average weights each annotation class equally whereas the macro-average weights each document equally, regardless of how many annotations are found in the document. Thus, macro-averaged results provided a straightforward way to compute statistical significance.

The lists of false negative results (i.e. gold standard annotations missed by the automatic system) and false positive predictions (i.e. predictions that did not have a match in the gold standard) provided the team with contextualized examples of the most frequent misclassifications made by their system. For example, in the prediction report shown in Figure 6, the term ‘alkyl’ was at the top of the false negatives of a prediction run for the CEMP task, with a total of 66 missed occurrences. Conversely, the document EP2610263A1 was the document with more incorrect positive predictions, with a total of 34 false positives. So, participants could use Markyt prediction reports to keep track of the mistakes being committed by their automatic systems, gain an understanding about what motivated the incorrect predictions and work on possible solutions.

There were no restrictions to the number of prediction analyses that a participant could run in Markyt during the challenge. In the final submission, each participant could submit a maximum of five runs per task and micro-averaged statistics were used to determine the top-scoring run for each team (the best F-score results). Furthermore, Markyt analytical environment enabled an overall view of misclassifications by all the systems for the CHEMDNER organizers. Specifically, Markyt showed the false positives and false negatives common to most systems and exposed the most ‘difficult’ documents, i.e. enabled further understanding of where/when the predictive ability of the automatic systems is more limited (Table 1). This information is considered valuable to better assess the difficulties that the automatic processing of chemical patents presents to those currently developing chemical recognition systems, and the capabilities of existing systems. Such insight will help prepare future editions of the CHEMDNER challenge and is believed to be of added value to the participating team (a macroscopic observation of each system’s prediction scores). Further details on this analysis can be found at the challenge overview paper (11, 12).

**Table 1.** **** Top document mismatches and term mismatches for final CEMP team submissions

	Top false negatives	Avg(FN) per run	Top false positives	Avg(FP) per run
Documents	EP2033959A1	19	EP2033959A1	53
	EP2610263A1	13	EP2546253A1	49
	EP2546253A1	13	EP2610263A1	47
	DE102004060041A1	8	DE102004060041A1	36
	US20100184776	4	US20090170813	21
Terms	Acid	33	Sodium	42
	Soy isoflavone	10	Alkyl	29
	Fibroblast	7	Ester	29
	Docetaxel	6	Opioid	28
	Aromatic or	4	Calcium	25
	heteroaromatic	4		

### Post-workshop software benchmarking

After the challenge, the results of the top-scoring run for each team, including recall, precision, and F-score for the best (micro-averaged) run of each system, were made public (11, 12). In particular, Markyt prediction analysis environment is now open to anyone who wishes to compare the performance of his system to CHEMDNER results.

**Figure 7.**
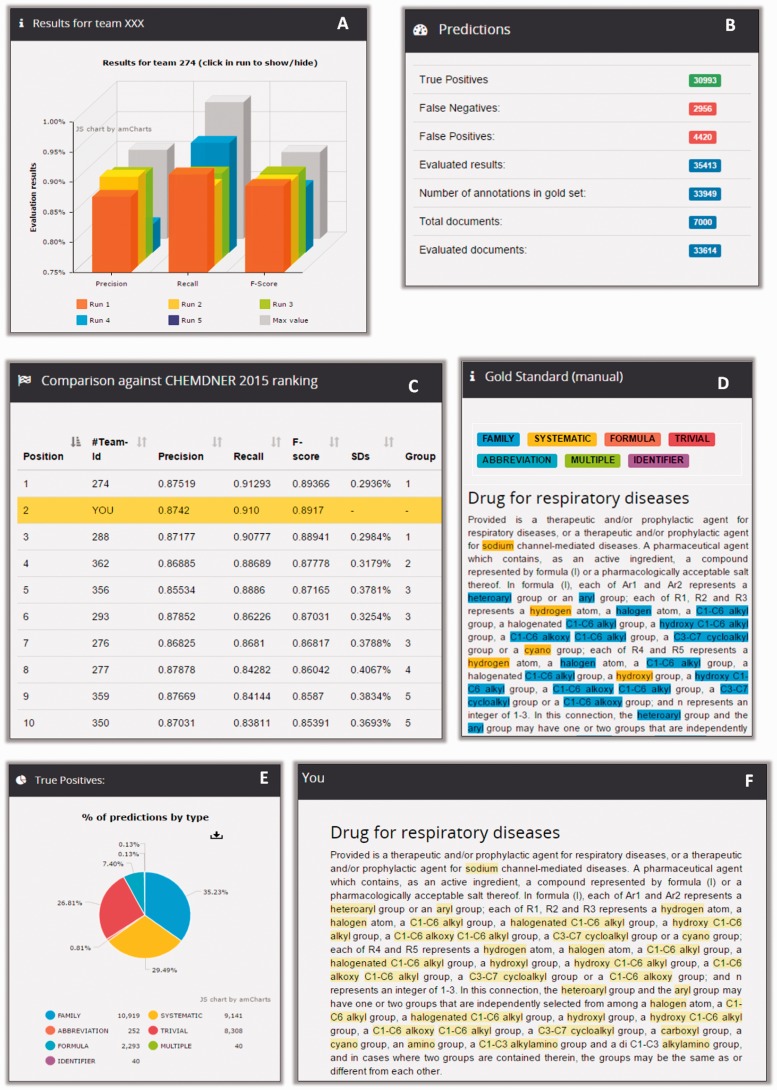
Example of CEMP post-workshop benchmarking in Markyt. New predictions on CEMP test set can be compared against the best predictions obtained during the competition (A) and the system is ranked accordingly (C). Also, general prediction statistics (B) can be explored per class type (E), looking into document matches and mismatches (D and E).

To further evaluate the significance of the difference between system performances, results were submitted to a bootstrap resampling. As exemplified in Figure 7, the evaluation tables of the CEMP and GPRO tasks depict the precision, recall and F-score of the best run of each team as well as illustrate the position of each team are covered within two standard deviations (SDs). When running a new prediction analysis at Markyt, the tool produces the usual performance statistics and compares these to the performance statistics of the systems that originally participated in the challenge. Therefore, developers receive in-depth information of their system’s predictive abilities against CHEMDNER gold standards and existing systems.

### Benefits of using Markyt

Table 2 summarizes the functionalities made available to the three user types of BioCreative/CHEMDNER, i.e. challenge organizer, text miner and expert annotator, in support of the main usage scenarios presented during the competition and after the workshop. Most notably, Markyt supports post-workshop system evaluation against the best performing systems in the competition.

**Table 2.** **** Main features of Markyt available to BioCreative/CHEMDNER and post-workshop users

User type	Role	Features
Challenge organizer	Competition management	Management of multi-user and iterative annotation projects Annotation quality control and consensus analysis Exportation of annotation sets (inline and stand-off formats) Publication of competition tasks Evaluation and ranking of final team submissions
CHEMDNER participant or text miner	System tuning and challenge submission	Prediction evaluation against various gold standards Validation of submission compliance with CHEMDNER format Identification of top prediction-annotation mismatches by document and type Visualisation of annotation mismatches Submission of final predictions
	Post-workshop benchmarking	Post-workshop evaluation against CHEMDNER resources (corpora and challenge scores)
Expert annotator	Manual curation	Highlight all occurrences of selected text fragment in document (or page of documents) Automatic annotation of a text fragment across all documents Remember last annotation type for new annotations Remember last annotation metadata for new annotations (e.g. last database id) Search by annotation Auto saving to prevent data loss
	Annotation revision	Visual differentiation of manual and automatic annotations to facilitate curation Download suggested terms to review, indexed by document Implicit acceptance of automatic annotations to speed up curation Batch change of annotation type for all annotations of a given term and type Delete all annotations of a given term (type-specific or not)

## Conclusion

Creating gold standards and enabling the controlled comparison of automatic prediction systems are key steps to keep improving the performance of automatic prediction systems in practical biomedical scenarios. Here, we described the Markyt Web-based platform for the visualisation, prediction and benchmark of chemical and gene entity recognition at the BioCreative/CHEMDNER challenge. This platform supported the preparation of the annotated document sets and, in particular, provides a semi-automatic curation workflow to improve the quality of the post-workshop test set (11, 12). Furthermore, Markyt allowed developers to test their predictions against the different CHEMDNER corpora and, more recently, to compare the performance of their systems with CHEMDNER final system ranking. The ultimate purpose was to provide computational support to challenge organizers and participants, and to make the resources and evaluation methods of BioCreative/CHEMDNER challenge readily available to the text mining community. Previous BioCreative tasks did not address the visualisation aspect sufficiently. Markyt bridged this gap by helping to prepare the annotated sets (targeting the needs manifested by human annotators) and enabling the analysis of prediction-annotation mismatches (helping text miners understand where/when the automatic systems tend to fail). Likewise, and although BioCreative related resources and tools are publicly available, no platform provided support to post-workshop benchmarking, namely, the development of new systems and the development of the participating systems.

Markyt has an open and general purpose architectural design that allows the integration of new subsystems or the modification of existing subsystems by third-parties. Markyt is also domain/application agnostic, notably it handles main document formats (TXT, HTML and XML), allows the customisation of annotation types and annotation metadata (e.g. database identifiers), and enables the customisation of quality monitoring (e.g. evaluation over one or several rounds of annotation, and looking into different metrics). Hence, Markyt has the potential of being adapted to other text mining tasks, including challenges or benchmark projects, annotation projects and database curation.

Finally, and thanks to the feedback received through the challenge, Markyt subsystems are being improved and they will be part of a new community-geared metaserver evaluation system. This innovative system will support the work of participants in upcoming editions of BioCreative/CHEMDNER as well as provide broader benchmarking and annotation services for text miners, database annotators and anyone that wishes to perform system benchmarking. Furthermore, the development of such novel community-geared metaserver will be under the umbrella of the OpenMinTeD project (http://openminted.eu/), the European open mining infrastructure for text and data, which will certainly help to identify and accommodate the requirements and concerns of different user communities as well as promote the use of the system worldwide.
